# Exophtalmie inflammatoire révélant un kyste hydatique orbitaire

**DOI:** 10.11604/pamj.2013.15.102.3088

**Published:** 2013-07-16

**Authors:** Zouheir Hafidi, Rajae Daoudi

**Affiliations:** 1 Université Mohammed V Souissi, service d'ophtalmologie A de l'hôpital des spécialités, Centre hospitalier universitaire, Rabat, Maroc

**Keywords:** Exophtalmie unilatérale, globe oculaire, œil, orbitotomie

## Image en médecine

A.L âgé de 65 ans, présentant une exophtalmie unilatérale droite, d'apparition brutale évoluant depuis sept mois. A l'examen on note une exophtalmie non axile de l’œil droit (globe dévié en bas et en dedans) avec un important œdème palpébral et une ophtalmoplégie complète. L'acuité visuelle était réduite à une perception lumineuse. Le segment antérieur était normal en dehors d'une kératite ponctuée superficielle avec un chémosis et une hyperhémie conjonctivale diffuse. L'examen du fond d’œil montrait une hyperhémie papillaire avec présence de quelques plis rétiniens. L'examen général était par ailleurs sans particularités. Un scanner orbito-cérébral réalisé en coupes axiales et coronales avec injection de produit de contraste confirmait l'absence d'anomalies du globe oculaire et mettait en évidence une formation ovalaire au niveau de l'orbite droite dans sa partie supéro-externe, de densité liquidienne très en faveur d'un kyste hydatique. Les examens à la recherche d'une deuxième localisation hydatique étaient négatifs en particulier la radiographie du thorax et l’échographie abdominale. L'abord chirurgical du kyste hydatique s'est fait par une orbitotomie latérale avec dépose du pilier osseux frontozygomatique. L'introduction d'une sonde de folley entre la paroi du kyste et sa coque et l'injection du sérum salé hypertonique a facilité l'accouchement du kyste dans sa totalité. Le diagnostic était confirmé par l'examen macroscopique et histologique de la pièce opératoire. Les suites opératoires étaient bonnes. L'exophtalmie a considérablement régressé cliniquement. Cependant, l'acuité visuelle ne s'est pas améliorée et le patient a gardé une limitation de l'oculomotricité dans toutes les directions.

**Figure 1:**
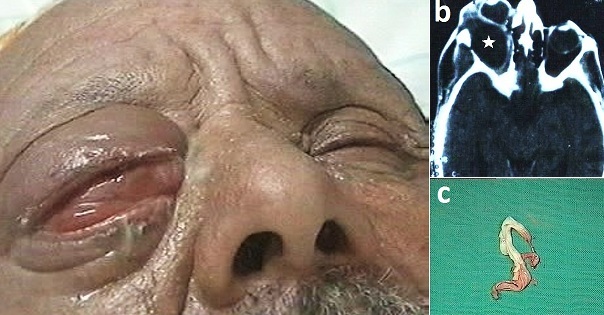
(a) exophtalmie non axile de l'œil droit (globe dévié en bas et en dedans) avec un important œdème palpébral. (b) scanner orbito-cérébral en coupes axiales mettant en évidence une formation ovalaire de densité liquidienne au niveau de l'orbite droite (étoile). (c) aspect macroscopique de la membrane proligère

